# Association between Precipitation and Diarrheal Disease in Mozambique

**DOI:** 10.3390/ijerph15040709

**Published:** 2018-04-10

**Authors:** Lindsay M. Horn, Anjum Hajat, Lianne Sheppard, Colin Quinn, James Colborn, Maria Fernanda Zermoglio, Eduardo S. Gudo, Tatiana Marrufo, Kristie L. Ebi

**Affiliations:** 1 Department of Epidemiology, University of Washington, 1959 NE Pacific Street, P.O. Box 357236, Seattle, WA 98195, USA; lindsaymhorn@gmail.com (L.M.H.); anjumh@uw.edu (A.H.); 2 Department of Environmental and Occupational Health Sciences, University of Washington, 1959 NE Pacific Street, P.O. Box 357234, Seattle, WA 98195, USA; sheppard@uw.edu; 3 Department of Biostatistics, University of Washington, 1959 NE Pacific Street, P.O. Box 357232, Seattle, WA 98195, USA; 4 United States Agency for International Development (USAID 1300 Pennsylvania Ave NW, Washington, DC 20004, USA; cquinn@usaid.gov; 5 Clinton Global Health Initiative, 383 Dorchester Ave., Suite 400, Boston, MA 02127, USA; jmcolborn@gmail.com; 6 Chemonics International, 1717 H St NW # 1, Washington, DC 20006, USA; fzermoglio@chemonics.com; 7 Instituto Nacional de Saude, Av Eduardo Mondlane, 1008, 2nd Floor, P.O. Box 264, Maputo, Mozambique; esamogudojr@gmail.com (E.S.G.); ttn.marrufo@gmail.com (T.M.); 8 Department of Global Health, University of Washington, 1959 NE Pacific Street, P.O. Box 357965, Seattle, WA 98195, USA

**Keywords:** climate change, diarrheal disease, Mozambique, precipitation, temperature

## Abstract

Diarrheal diseases are a leading cause of morbidity and mortality in Africa. Although research documents the magnitude and pattern of diarrheal diseases are associated with weather in particular locations, there is limited quantification of this association in sub-Saharan Africa and no studies conducted in Mozambique. Our study aimed to determine whether variation in diarrheal disease was associated with precipitation in Mozambique. In secondary analyses we investigated the associations between temperature and diarrheal disease. We obtained weekly time series data for weather and diarrheal disease aggregated at the administrative district level for 1997–2014. Weather data include modeled estimates of precipitation and temperature. Diarrheal disease counts are confirmed clinical episodes reported to the Mozambique Ministry of Health (*n* = 7,315,738). We estimated the association between disease counts and precipitation, defined as the number of wet days (precipitation > 1 mm) per week, for the entire country and for Mozambique’s four regions. We conducted time series regression analyses using an unconstrained distributed lag Poisson model adjusted for time, maximum temperature, and district. Temperature was similarly estimated with adjusted covariates. Using a four-week lag, chosen a priori, precipitation was associated with diarrheal disease. One additional wet day per week was associated with a 1.86% (95% CI: 1.05–2.67%), 1.37% (95% CI: 0.70–2.04%), 2.09% (95% CI: 1.01–3.18%), and 0.63% (95% CI: 0.11–1.14%) increase in diarrheal disease in Mozambique’s northern, central, southern, and coastal regions, respectively. Our study indicates a strong association between diarrheal disease and precipitation. Diarrheal disease prevention efforts should target areas forecast to experience increased rainfall. The burden of diarrheal disease may increase with increased precipitation associated with climate change, unless additional health system interventions are undertaken.

## 1. Introduction

Sub-Saharan Africa is projected to be particularly affected by climate change [1]. Climate variability and change present current and future risks to human health in this region, where many countries have high exposure to climate-related hazards, as well as low capacity to manage the associated risks [2]. Increases in temperature and precipitation intensity are already occurring because of climate change [3] (pp. 1–19). Changes in precipitation and temperature not only alter the geographic range, pathogenicity, seasonality, and survival of disease-causing pathogens but may also increase human exposure and jeopardize the infrastructure necessary to prevent disease transmission [4]. Diarrheal diseases, already of significant concern in sub-Saharan Africa, are amongst a wide range of health outcomes sensitive to weather and climate. Kolstad and Johansson [5] estimated that by the end of the 21st century, climate change might increase the relative risk of diarrhea in Southern Africa by more than 20 percent.

Transmission pathways through which precipitation increases diarrheal diseases are broad and complex, and rainfall variability can influence diarrheal disease in many ways. Flooding, often due to heavy precipitation, is linked to increased diarrheal disease prevalence [6]. Rainfall runoff and flooding can lead to human exposure to pathogens by flushing pathogens from environmental reservoirs or fecal matter into freshwater supplies [7,8,9,10]. In contrast, water scarcity can necessitate consumption of unsafe water, as well as decrease hygienic practices, increasing diarrheal disease [11]. Studies in low-resource settings in Africa found an increased number of cases associated with both the dry and wet seasons [1,12,13], below average rainfall [11], heavy rains [14], and rainfall shocks (deviations from the long-term average) [15].

Increased temperature has been associated with increased diarrheal disease, as warmer temperatures may cause increased pathogen proliferation in food and water sources [9,16]. Carlton et al.’s global systematic review of temperature and diarrheal disease found a seven percent increase in all-cause diarrheal disease for each degree Celsius increase in temperature [4]. Bandyopadhyaya et al.’s examination of temperature and childhood diarrhea in 14 sub-Saharan African countries found a one degree Celsius increase in the average maximum temperature to increase diarrhea prevalence by one percent [11]. 

Although diarrheal diseases are considered a leading cause of morbidity and mortality in Africa, the quantity of evidence examining the association between climate and these diseases in sub-Saharan Africa is low [17]. The consistent collection of long-term disease data required to perform such analyses is resource intensive and challenging in less developed countries and settings with weak health infrastructure [1]. As such, many existing studies in sub-Saharan Africa focused more broadly on seasonal trends and annual disease peaks of all-cause diarrhea [7,12,13,14,18]. Studies that estimated the direct association primarily relied on smaller geographic areas or shorter timescales [15].

Mozambique is a country with over seven million counts of diarrheal disease episodes from all public facilities in the country reported between 1997 and 2014, in a population of 28.0 million in 2015 [19]. Diarrheal disease was Mozambique’s fifth leading cause of death, as well as its fourth leading cause of death and disability combined in 2015 [19]. No prior study examined the relationships between weather variables and diarrheal disease in Mozambique. 

Using a rich, 18-year data source spanning the country of Mozambique, we estimated the short-term association between rainfall and diarrheal disease at national and regional levels. As a secondary analysis, we examined the association between temperature and diarrheal disease. The data were collected at the weekly time scale, a high resolution that reduces the potential to miss critical disease fluctuations following rain, heat, or cold events. Risk estimates can inform improved policies and programs to support the prevention of diarrheal disease.

## 2. Materials and Methods

Mozambique is a country situated on the southeastern coast of Africa with a tropical climate characterized by generally hot and rainy summers (November to April), where temperatures in parts of the country may average over 35 °C and cooler and dry winters, and where monthly mean minimum temperatures may drop below 20 °C [20]. In 2015, the estimated life expectancy was 54 years for males and 60 years for females [19]. Under-five mortality decreased steadily in recent decades, dropping from 2600 to 1600 per 100,000 females and 2800 to 1800 per 100,000 males between 1990–2015 [19]. The 2015 Human Development Index ranks Mozambique 180th out of 188 countries and territories [21]. Political instability, historic floods and droughts, and increasing malnutrition and stunting have likely contributed to development setbacks. 

### 2.1. Health Data

We conducted an ecologic study using weekly aggregated disease count data from more than 141 administrative districts in Mozambique from 1997–2014. Diarrhea is defined as the elimination of feces more liquid than normal and more than 3 times a day. Counts are confirmed clinical episodes as diagnosed by clinicians, including nurses and doctors. Data come from all public facilities in the country. Private facilities do not report through Weekly Epidemiological Bulletin, or Boletim epidemiological seminal (BES), though usage of private facilities is quite low in the country.

Case counts were tabulated at the clinics using a simple form to mark disease seen on a given day. Counts were aggregated weekly at the district administrative level for reporting to province, and finally on to the Mozambique Ministry of Heath reportable disease registry. Although data were available beginning in 1989, we chose this time period because, on average, completeness of reporting improved from 1997 forward (see Supplemental Figure S1). Districts that provided no case report for a given week were considered missing. 

The number of districts varied over the 18 years of follow-up; several districts were abolished, two split, and more than 10 were formed. We included individual districts in the analysis if they reported ≥85 percent of possible weeks. As a result, we excluded four districts from the analysis, three of which appeared to begin reporting midway through follow-up and may have been newly created.

### 2.2. Weather Data

Precipitation data were modeled estimates obtained from the Climate Hazards Group InfraRed Precipitation with Stations (CHIRPS) dataset, which spans nearly worldwide with data collection beginning in 1981 [22]. The CHIRPS dataset contains daily rainfall data derived from a combination of satellite-derived precipitation estimates or merged satellite data, model re-analysis data for large areas, and weather station rainfall data gridded to 0.05 × 0.05 degree spatial resolution. CHIRPS data are available daily for the period 1981–2014 and aggregated to the weekly level by the Climate System Analysis Group (CSAG). 

Precipitation, or ‘wet days’, was defined as the number of days it rained one millimeter (mm) or more in a week. This classification is one of 27 indices developed by the World Meteorological Organization Commission for Climatology and the Expert Team on Climate Change Detection and Indices (ETCCDI) [20]. 

Temperature data come from the Climate Research Unit (CRU), which utilizes more than 4000 global weather stations [23]. The CRU dataset was developed and is maintained primarily by the United Kingdom’s Natural Environment Research Council and the United States Department of Energy [24]. The data consist of weekly time series estimates of multiple temperature variables as far back as 1979, and are gridded to 0.05 × 0.05 degree spatial resolution. 

### 2.3. Statistical Analysis

We conducted a time series analysis and fit a generalized linear model (GLM) assuming an overdispersed Poisson model to estimate short-term (i.e., less than seasonal, or weekly) associations between weekly case counts of diarrheal disease and number of wet days (precipitation > 1 mm) per week [25,26]. Informed by prior research, we chose a priori to lag our wet day variable up to four weeks using an unconstrained distributed lag model with multiple lagged weeks of precipitation simultaneously in the model [27]. The four-week lag was selected to allow for pathogen incubation, illness presentation, and the subsequent clinical visit required to be included as a case, and was central among a range found in existing literature [1,8,10,14]. 

Time (year), temperature (degrees Celsius), and region/district were covariates included in the analysis to control for potential confounding. We controlled for seasonality and long-term trends using a cubic smoothing spline for time with four knots per year (degrees of freedom (df) = 72). We controlled for same-week (lag 0) district-level maximum temperature using cubic splines with knots placed every 5 °C [8,16,28]. In addition to time and temperature, we included a regional indicator variable in our national model, owing to the country’s spatial variation in disease burden and precipitation. 

Concerned that our national estimate masks sizeable heterogeneity in both diarrheal disease burden and precipitation across Mozambique, we also estimated regional associations using region-specific time-series data. Region-stratified models similarly controlled for time and temperature, but also included an indicator variable for the district. Four regions (northern, coastal, central, and southern) are defined in part by province boundaries and in part by climate relationships.

Results are presented as the estimated percent increase and 95% confidence intervals (CI) in disease counts associated with one additional wet day at a four-week lag, adjusted for effects at one, two, and three weeks using an unconstrained distributed lag model.

As secondary analyses, we fit similar models to estimate the association between maximum temperature (week’s maximum degrees Celsius) and case counts of diarrheal disease. These models were fit unlagged at the national and regional level, controlling for time, region/district, and precipitation using a cubic spline for the number of wet days.

### 2.4. Sensitivity Analysis

We performed two sensitivity analyses to determine whether our results were robust to choices made in model fitting at the national level. We changed the degree of smoothing in both the temperature and time splines by halving and doubling the number of knots in each (i.e., 2 and 8 knots per year for time and 2.5 and 10 °C for temperature), and compared our risk estimates to those four models. In addition, we performed sensitivity analysis to explore different precipitation time lags, which were between 0 and 8 weeks.

## 3. Results

### 3.1. Precipitation

**Figure 1:**
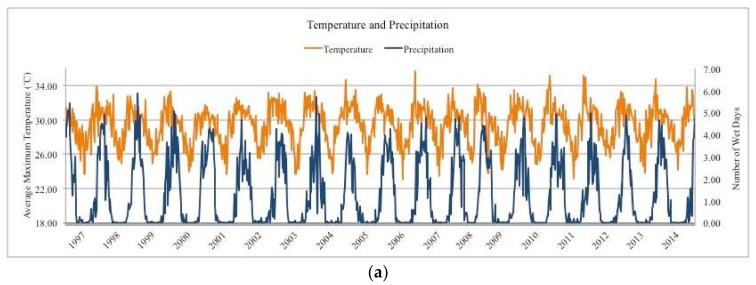

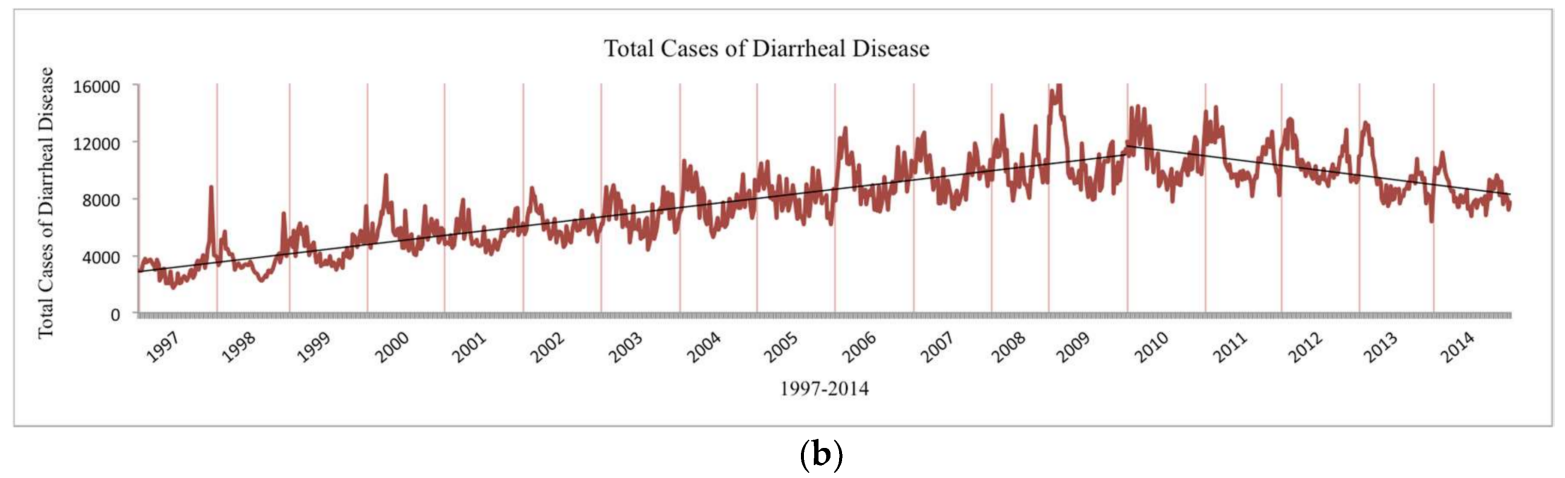
Temporal variation in climatic variables and diarrheal disease in Mozambique, 1997–2014, among all 141 administrative districts. (**a**) Temperature (orange, *x*-axis) and precipitation (blue, *y*-axis) defined as the number of wet days (precipitation < 1 mm per week) by week of follow-up; (**b**) Total number of cases in Mozambique by week of follow-up.

Nationally, there was an average of 1.23 wet days per week, and 1.62, 1.29, 1.01, and 0.82 in the northern, central, coastal, and southern region, respectively. Weather variables displayed pronounced seasonality nationally (Figure 1) and regionally (Figure S2) and corresponded with the country’s historical climate profile of wet summer months (December, January, and February) and dry winter months (June, July, and August) [20].

Climate profiles varied regionally (Table S1); the southern region received appreciably less rainfall than the other regions across all precipitation indicators. It also had the largest range in temperatures, with the highest average maximum temperatures and lowest average minimum temperatures. 

### 3.2. Diarrheal Disease

There were 7,315,738 reported cases of diarrheal disease over 18 years from 141 administrative districts (*n* = 126,056). Total and weekly average cases varied by region (Table 1). The number of cases reported weekly increased from 1997–2009, and case counts peaked nationally and within all four regions in 2009. There was a decreasing trend in the number of cases reported weekly from 2009–2014. There was an average of 85 cases reported by each district every week (Figure 1 and Table S2).

Nationally, weekly case counts revealed marked seasonality (Figure S3). Regionally, seasonality was more pronounced in the northern and central regions (Figure S4). Average diarrheal disease case counts peaked between weeks four and 12, which correspond to the weeks at the end of and following the wet season, when the heaviest rainfall and tropical cyclones typically occur [20]. Case counts dipped in the cooler, dry winter months.

### 3.3. Risk Estimates—Precipitation

**Figure 2:**
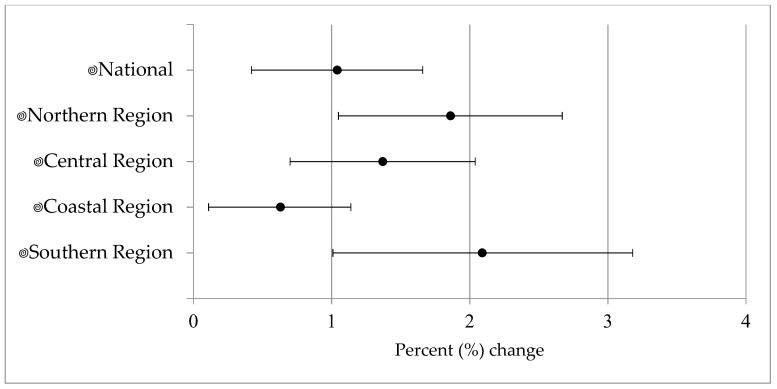
National and regional percent increase and 95% confidence interval in diarrheal disease associated with one additional wet day at a four-week lag, adjusted for effects at one, two, and three weeks using an unconstrained distributed lag model. The national model was adjusted for time, temperature, and region, while the regional model was adjusted for time, temperature, and district.

We estimated a 1.04% (95% CI: 0.42, 1.66) increase in diarrheal disease counts for each additional wet day considered over a consecutive four-week period, controlling for time, average high temperature, and region (Figure 2). Regionally, one additional wet day (over 4 week lag) was associated with an increase in disease of 1.86% (95% CI: 1.05, 2.67), 1.37% (95% CI: 0.70, 2.04), 0.63% (95% CI: 0.11, 1.14), and 2.09% (95% CI: 1.01, 3.18) in the northern, central, coastal, and southern region, respectively (Figure 2). The southern and northern regions had the strongest association with precipitation. 

### 3.4. Risk Estimates—Temperature

Nationally, we estimated a 3.64% (95% CI: 3.35, 3.93) increase in diarrheal disease for each one degree Celsius increase in the hottest day of the concurrent week. Regionally, each one degree Celsius increase in maximum temperature was associated with a 1.45% (95% CI: 0.77, 2.13), 1.87% (95% CI: 1.44, 2.30), 5.74% (95% CI: 5.18–6.29), and 2.15% (95% CI: 1.51, 2.80) increase in diarrheal disease in the northern, central, coastal, and southern regions, respectively (Table 2). The coastal region, while least impacted by increased precipitation, had the strongest association with an increase in temperature. 

### 3.5. Sensitivity Analysis 

Model estimates were robust to both halving and doubling of the number of knots controlling for temperature (Table S3). However, estimates were sensitive to changes in the control of time. We estimated an increased effect size of 1.55% (95% CI: 0.97, 2.14) by halving the number of knots (df = 2 × 18 = 36) and a decreased effect size of 0.58% (95% CI: −0.05, 1.20) by doubling the number of knots (df = 8 × 18 = 144). The estimated effect size decreased progressively when the model was fit with one to four knots per year and appeared to level off at ≥five knots per year (Figure S5). Lag exploration revealed that precipitation was most strongly associated with disease at four weeks (Figure S6).

## 4. Discussion

Our results indicate that in Mozambique, precipitation is positively associated with diarrheal disease. This association was found nationally and in all four regions, to varying degrees, with stronger associations in northern and southern regions. Overall, we estimated a 1.04% (95% CI: 0.42, 1.66) increase in disease for every additional wet day in a week, four weeks prior, and regionally this was as high as a 2.09% (95% CI: 1.01, 3.18) as observed in the southern region. 

Increased diarrheal disease in the weeks following a precipitation event or temperature increase represents a burden on health systems to treat these additional cases. Although a few percent increase is relatively small, this can create undue burden in low resource settings. Further, using weekly instead of daily data and with uncertainties about the weather data, the reported association could be an underestimate of the true effect. These analyses suggest that without implementing additional interventions, the decreasing trend in the number of cases of diarrheal disease may level off (as indicated by our data); however, there is a distinct possibility that cases will increase over the coming decades, assuming climate change alters precipitation patterns, resulting in an increase in the number of wet days. The magnitude and pattern of future burdens will depend on changes in weather and climate in the four regions of Mozambique, the rate of population increase, the effectiveness of efforts to increase access to safe water and improved sanitation, the effectiveness of adaptation, and other interventions to prevent contamination of food and water with disease-causing pathogens [29]. 

Although research has shown diarrheal diseases to be affected by weather; studies revealed pronounced heterogeneity in the association between all-cause diarrhea and precipitation. Heterogeneous associations could be due to differing pathogen burdens in other countries or methodological issues such as less granular monthly time series data, the limited time frame in which these studies spanned, or the limited geographic region of prior studies. The positive association found here aligns with Alexander and colleagues’ finding that rainfall was positively associated with the subsequent month’s diarrheal disease in Botswana [1]. 

In Mozambique, the majority of the population does not have access to adequate sanitation facilities; in rural areas, most of the population practices open defecation with no hygienic separation of fecal matter from human contact [30]. Even for the 42 percent of urban dwellers in Mozambique with access to adequate sanitation, heavy rain events can overflow sewer systems and contaminate drinking water [30]. These conditions are conducive to flooding and heavy precipitation events that flush fecal matter into areas where humans may become exposed.

Our exploratory results were consistent with existing research that found a positive association between temperature and diarrheal disease. Though the coastal region’s disease burden had the smallest association with an increased precipitation, a one degree Celsius increase in temperature was associated with a nearly six percent increase in disease. A possible explanation is that replication rate and transmission cycle of coastal region’s causative pathogens are more sensitive to temperature. However, we must interpret these results with caution, as they are purely exploratory.

Our study had several strengths. First, our data span the entire country and were not limited to a specific village or district. Second, 18 years of time series data at the weekly resolution is rare, especially in a sub-Saharan African country where health infrastructure is often weak and data collection may be inconsistent [1]. Weekly aggregated data allow us to estimate fine temporal associations. Third, we estimate direct associations between weather and diarrheal disease, whereas many existing studies have focused on trends or seasonal disease peaks. 

Our study has several limitations. Common to ecological studies is unmeasured confounding. Our study may include year-to-year variation in population, trends in the number of reporting health clinics over the years of follow-up, and access to improved sanitation or safe water. We adjusted for time as a proxy for various unmeasured confounders that may vary over time, but for which we do not have information [25]. As our sensitivity analysis indicated, our estimates were sensitive to how time was specified in the model, confirming that time was indeed a strong confounder in this analysis, and highlighting a common concern in time series analysis: that results may be sensitive to model choices. We felt that four knots per year was sufficient to remove confounding effects of seasonality in a sensible way without overcontrolling. 

While our diarrheal disease-count data are described above as “confirmed clinical cases”, they do not contain person-level identifiers, so repeat visits for a given disease episode may be included. Therefore, the term “case counts” must be interpreted accordingly. It is assumed that short term variation in reported counts will reflect variation in disease incidence and not variation in proportions of repeat visits. However, underreporting of diarrheal disease is ubiquitous. Aggregated health clinic counts only include individuals who sought medical care for their illness and who were subsequently reported by the clinic. It would not capture people without the means or access to care, those with mild symptoms, or people who were not captured following their clinic visit, as evidenced by low reporting rates prior to 1997 (Figure S1). Individual-level factors may influence health-seeking behavior, such as education, demographics variables, social status, economic resources, and religious beliefs, and could potentially impact results. However, we may be less concerned with bias from individual-level characteristics, given that our unit of analysis is the district rather than individual [25].

## 5. Conclusions

Climate variability and change present current and future risks to human health. Low-resource settings, such as sub-Saharan Africa, are expected to experience larger increases in the burden of diarrheal disease with climate change, because these regions will, in many cases, have higher exposure to climate-related hazards, such as extreme precipitation or temperature events, and because these regions have low capacity to manage such risks. Africa is particularly vulnerable, because it is already facing weather conditions conducive to the spread of diarrheal disease that climate change is expected to exacerbate [6].

This study is an important first step towards understanding climate-drivers of diarrheal disease in Mozambique and supporting the development of an early warning system to improve health system preparedness and response. These additional cases of diarrheal disease are potentially preventable using the increasing skill in forecasting precipitation over seasonal timescales. Having advanced warning (e.g., an early warning and response system) that a week is expected to be wetter than normal would provide valuable time to put interventions in place, such as increasing access to oral rehydration in local health care centers and increasing education on appropriate use and handling of water (such as boiling drinking water) and on sanitation practices that can reduce transmission of diarrheal pathogens. Developing and deploying such an early warning system would increase population resilience to outbreaks of diarrheal disease over coming decades.

## Figures and Tables

**Table 1:** National and regional descriptive statistics. Observations are the total number of weeks reported over the study period.

Area	Observations (*n*)	Districts (*n*)	Total Diarrhea Cases	Mean (sd) Diarrhea Cases Per Week	Mean (sd) Wet Days Per Week	Mean (sd) Maximum Temp (°C)	Mean (sd) Minimum Temp (°C)
National	126,056	141	7,315,738	58.08 (80.86)	1.23 (1.91)	29.45 (2.94)	18.94 (3.46)
Region							
Northern	34,555	38	1,681,018	48.65 (66.69)	1.62 (2.29)	29.19 (2.59)	18.83 (2.99)
Central	32,113	37	1,956,881	60.94 (63.08)	1.29 (1.98)	29.35 (3.18)	18.09 (3.47)
Coastal	45,821	51	3,130,416	68.32 (104.74)	1.01 (1.62)	29.57 (2.97)	20.06 (3.28)
Southern	13,567	15	547,423	40.35 (43.69)	0.82 (1.26)	29.95 (2.97)	17.44 (3.91)

**Table 2:** Secondary analysis: Percent change (95% confidence interval) of diarrheal disease associated with a one degree Celsius increase in the concurrent week’s maximum temperature.

Area	% Change (95% CI)
National ^a^	3.64 (3.35, 3.93)
Northern Region ^b^	1.45 (0.77, 2.13)
Central Region ^b^	1.87 (1.44, 2.30)
Coastal Region ^b^	5.74 (5.18, 6.29)
Southern Region ^b^	2.15 (1.51, 2.80)

^a^ Model adjusted for time, precipitation and region; ^b^ Model adjusted for time, precipitation and district.

## Supplementary Materials

The following are available online at http://www.mdpi.com/1660-4601/15/4/709/s1, Figure S1: Annual diarrheal disease reporting percentages in Mozambique for the years 1989–2014, Figure S2: Regional seasonality of precipitation, Table S1: Mean values of weekly weather variables, nationally and by region, Table S2: Total observations (*n*), mean, and standard deviation of total cases reported each week nationally and regionally, by year, Figure S3: Average number of cases reported at the district-level each week, by week number, nationally, Figure S4: Regional seasonality of diarrheal disease in Mozambique, Table S3: Sensitivity analysis for the degree of smoothing in the temperature spline for the association between precipitation and diarrheal disease using the national model, Figure S5: Percent change (and 95% confidence intervals) of diarrheal disease associated with precipitation at the national level with increasing control of time, 0–12 knots per year (left to right). Figure S6: Percent change and 95% confidence intervals for diarrheal disease associated with precipitation at various lags of 0–8 weeks, controlling for time, average maximum temperature, and region.
