# Supplementary material for: Association between Precipitation and Diarrheal Disease in Mozambique
Source: Int J Environ Res Public Health. 2018 Apr 10;15(4):709. doi: 10.3390/ijerph15040709 (PMC5923751; doi:10.3390/ijerph15040709)
Supplement: Supplementary file 1 [file ijerph-15-00709-s001.pdf]

SUPPLEMENT

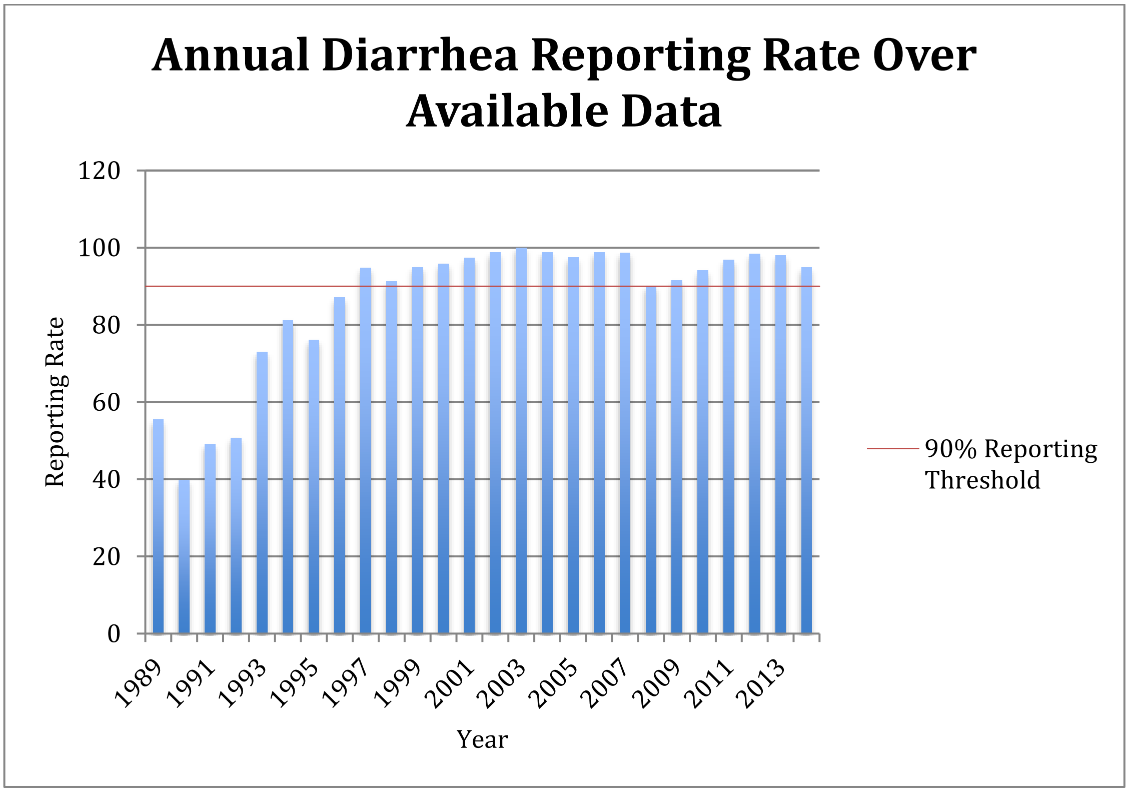

Figure S1. Annual diarrheal disease reporting percentages in Mozambique for the years 1989-2014, when data were available. The reporting rate is the percentage of weeks that disease counts were reported each year, out of all possible weeks among the districts. Weeks prior to 1997 were excluded from analyses due to incomplete reporting.

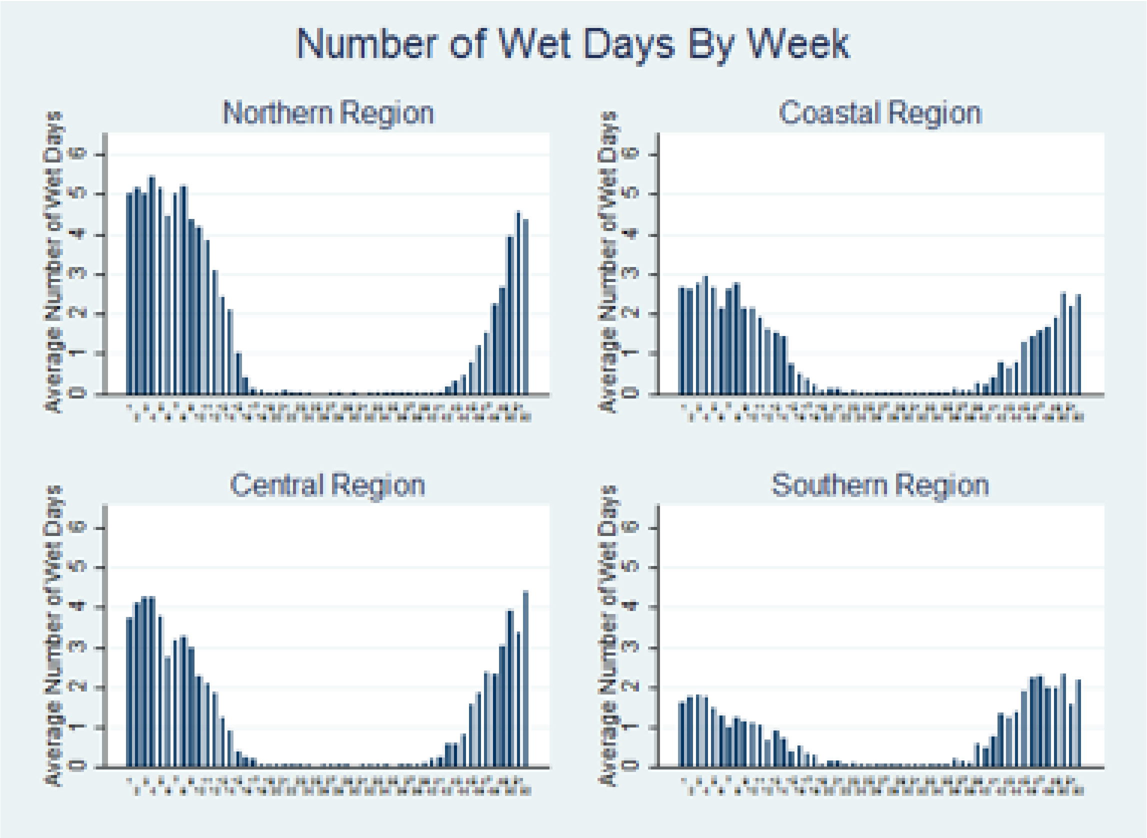

Figure S2. Regional seasonality of precipitation. Average number of wet days by week number, 0-52 (x-axis) in Mozambique's 4 regions.

Table S1. Mean values of weekly weather variables, nationally and by region. Green colors highlight the two lower values over the four regions. Orange colors highlight the two higher values over the four regions.

	Highest weekly maximum temperature	Lowest weekly maximum temperature	Highest weekly minimum temperature	Lowest weekly minimum temperature	Highest single-day rainfall volume (mm)	Total volume of rainfall during the week (mm)
National	32.50	26.39	20.58	17.21	8.47	17.59
Region						
Northern	32.60	26.08	19.98	16.13	8.58	18.63
Central	32.70	26.54	21.54	18.46	8.74	16.18
Coastal	31.43	26.78	20.19	17.36	8.50	20.87
Southern	34.34	25.61	19.71	15.11	7.10	11.39

Table S2. Total observations (n), mean, and standard deviation of total cases reported each week nationally and regionally, by year. Orange lines highlight mean size relative to other years and highest mean case counts are in red (all 2009).

	National		
Year	n	mean	sd
1997	6,812	24.54	59.75
1998	6,665	28.15	66.2
1999	6,910	33.69	68.16
2000	6,979	43.11	89.55
2001	7,100	39.78	68.7
2002	7,194	44.42	65.04
2003	7,155	49.1	81.5
2004	7,195	55.21	84.64
2005	7,096	59.8	76.53
2006	7,196	67.39	79.98
2007	7,184	69.57	76.18
2008	6,529	78.5	89.72
2009	6,726	84.81	97.74
2010	6,925	81.12	84.06
2011	7,107	78.18	89.33
2012	7,210	75.71	87.66
2013	7,177	68.43	72.85
2014	6,958	63.14	68.21

	Northern			Central		
Year	n	mean	sd	n	mean	sd
1997	1,906	12.42	14.96	1,703	28.69	48.04
1998	1,945	17.03	66.78	1,625	27.35	32.86
1999	1,952	18.03	24.36	1,770	37.31	69.56
2000	1,921	23.54	28.75	1,777	42.57	72.59
2001	1,948	25.33	34.48	1,768	43.67	62.32
2002	1,935	34.50	37.75	1,855	49.33	56.98
2003	1,941	38.27	48.64	1,836	51.75	78.83
2004	1,942	46.36	45.69	1,843	56.71	47.51
2005	1,948	52.37	77.92	1,817	64.71	65.96
2006	1,957	67.00	74.88	1,845	70.12	59.24
2007	1,955	64.50	61.59	1,863	71.26	55.30
2008	1,681	74.43	83.77	1,669	80.71	66.19
2009	1,838	80.26	87.00	1,634	85.60	69.33
2010	1,932	75.75	77.33	1,755	84.97	64.43
2011	1,946	70.43	84.07	1,808	79.10	58.93
2012	1,968	66.52	84.54	1,869	79.05	57.54
2013	1,957	60.18	69.49	1,873	73.04	56.01
2014	1,883	52.82	60.61	1,803	68.05	47.74

	Coastal			Southern		
Year	n	mean	sd	n	mean	sd
1997	2,462	33.96	88.06	741	14.91	19.40
1998	2,340	40.44	86.88	755	20.43	25.34
1999	2,473	47.07	91.35	715	21.21	31.18
2000	2,554	61.11	125.98	727	32.88	62.61
2001	2,612	52.63	93.40	772	23.85	25.20
2002	2,636	53.18	88.14	768	27.45	25.92
2003	2,603	60.48	106.75	775	31.77	37.03
2004	2,633	67.60	125.74	777	31.77	30.66
2005	2,571	67.22	89.03	760	42.03	35.14
2006	2,618	73.73	101.46	776	40.49	35.25
2007	2,607	77.66	101.09	759	50.67	43.74
2008	2,433	85.70	113.57	746	59.23	47.02
2009	2,461	93.48	123.94	731	60.27	53.24
2010	2,504	88.88	104.05	734	59.54	57.50
2011	2,597	89.21	114.36	756	58.08	51.35
2012	2,612	86.31	111.94	761	54.86	46.20
2013	2,589	76.26	89.15	758	51.59	46.69
2014	2,516	72.60	88.19	756	45.69	37.92

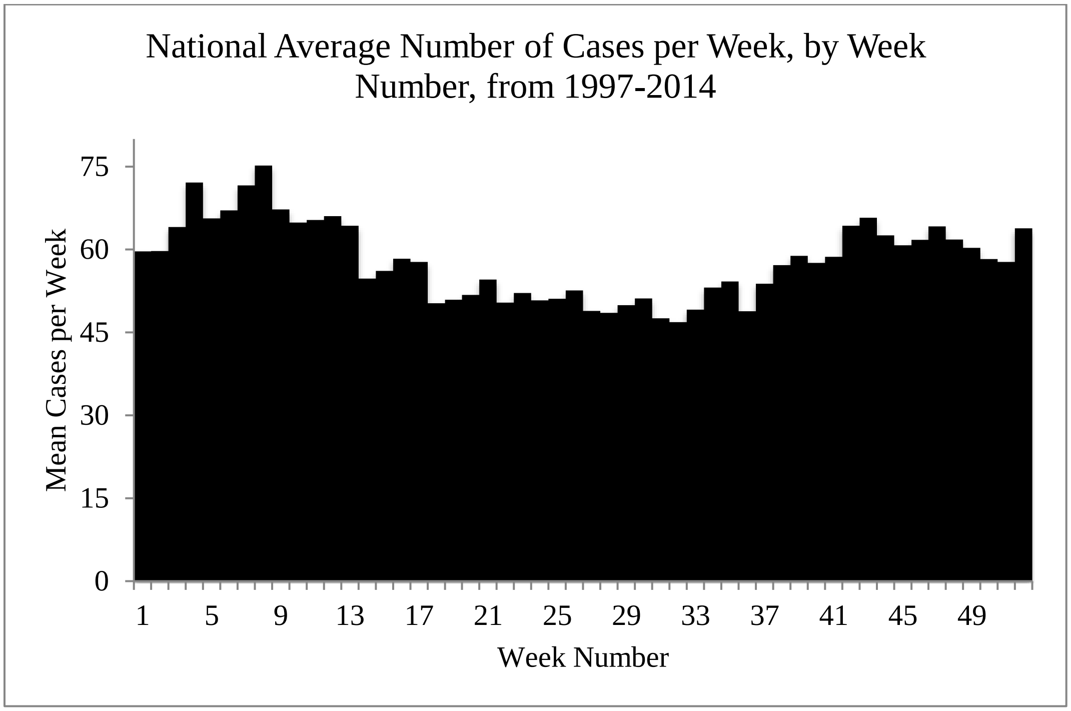

Figure S3. Average number of cases reported at the district-level each week, by week number, nationally.

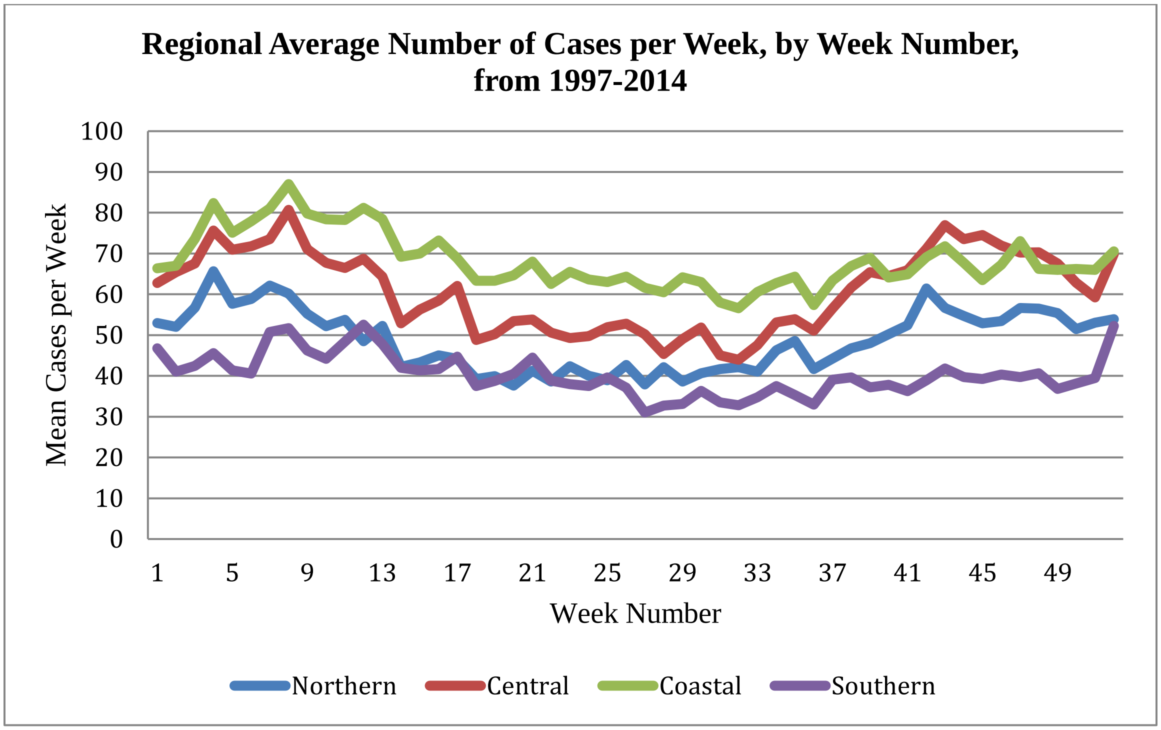

Figure S4. Regional seasonality of diarrheal disease in Mozambique. Mean diarrheal disease cases by week of year and region. We believe that the week 52 uptick seen in each region is due to extra days at the end of the year (>7) being counted as week 52.

~~**Table S3.** Incidence Rate Ratio (IRR) and 95% confidence interval for diarrheal disease associated with one additional wet day at various lags using an unconstrained distributed lag model. The national model was adjusted for time, temperature, and region.~~

	~~IRR (95% CI)~~
~~No lag~~	~~0.998 (0.991, 1.004)~~
~~1 week Lag~~	~~0.999 (0.993, 1.006)~~
~~2 Week Lag~~	~~1.001 (0.995, 1.008)~~
~~3 Week Lag~~	~~1.007 (1.00, 1.013)~~
~~4 Week Lag~~	~~1.01 (1.004, 1.017)~~

Table S34. Sensitivity analysis for the degree of smoothing in the temperature spline for the association between precipitation and diarrheal disease using the national model. Estimated percent increase and 95% CI for the final model fit with one knot per 5 degree Celsius change in temperature. Below that are model estimates for halving (one knot per 10˚C) and doubling (one knots per 2.5˚C) spline flexibility.

Model[a]	Percent Increase	95% CI LL	95% CI UL
Less Flexible *1 knot/10˚C*	1.08	0.46	1.70
Final Model *1 knot/5˚C*	1.04	0.42	1.66
More flexible *1 knot/2.5˚C*	1. 02	0.41	1.64

CI = 95% confidence interval; LL = lower limit; UL = upper limit

[a]Controlling for time, average high temperature and region

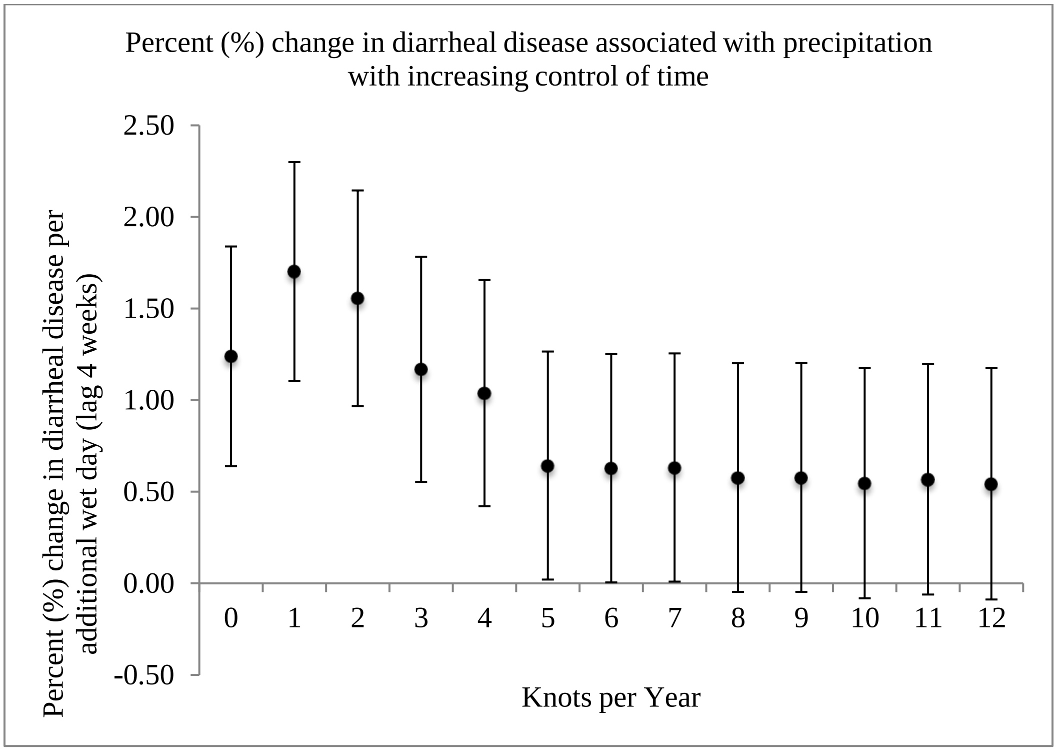

Figure S5. Percent change (and 95% confidence intervals) of diarrheal disease associated with precipitation at the national level with increasing control of time, 0-12 knots per year (left to right). As a sensitivity analysis, time is adjusted for with varying control by altering the number of knots per year. Estimates are sensitive to changes in time adjustment. All estimates control for time (varyingly), temperature, and region.

Consistent with prior research and to allow for pathogen incubation, illness presentation, and the subsequent clinical visit requirement to be included as a case count, it was decided *a priori* to lag the wet day variable four weeks. To test our *a priori* decision to use a four-week lagged association, we estimated percent changes (with 95% CIs) for lags from zero-eight weeks in our national model, controlling for time, average maximum temperature, and region (Figure 2). Consistent with existing studies, the four-week lag had the strongest association between wet days and diarrheal disease.

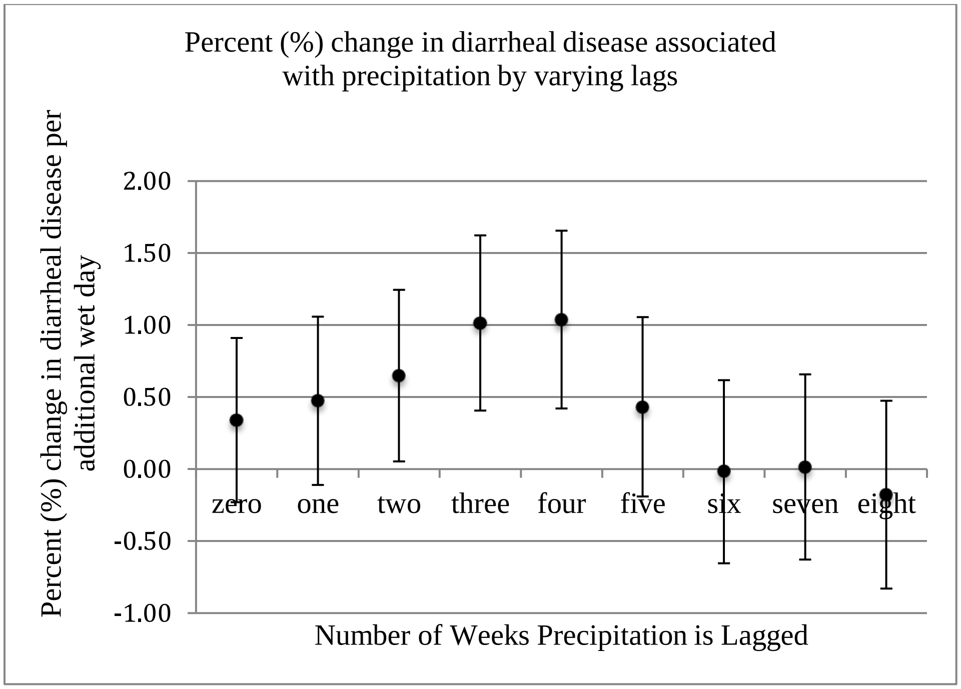

Figure S6. Percent change and 95% confidence intervals for diarrheal disease associated with precipitation at various lags of 0-8 weeks, controlling for time, average maximum temperature, and region.
